# The scope of empowerment for conservation and communities

**DOI:** 10.1111/cobi.14249

**Published:** 2024-03-15

**Authors:** Michael A. Petriello, Lauren Redmore, Aby L. Sène, Dhananjaya Katju, Lilian Barraclough, Sara Boyd, Carly Madge, Anastasia Papadopoulos, Reddi S. Yalamala

**Affiliations:** ^1^ The Center for Science and Society Columbia University New York New York USA; ^2^ Commission for Environmental, Economic and Social Policy (CEESP) International Union for the Conservation of Nature Gland Switzerland; ^3^ Community‐Engaged CoLab, School for Resource and Environmental Studies Dalhousie University Halifax Nova Scotia Canada; ^4^ Aldo Leopold Wilderness Research Institute, Rocky Mountain Research Station USDA Forest Service Missoula Montana USA; ^5^ Parks and Conservation Area Management Clemson University Clemson South Carolina USA; ^6^ Department of Environmental Science American University Washington, DC USA

**Keywords:** collaboration, community‐based conservation, conservation social science, disempowerment, empowerment, Global South, participation, positionality, ciencias sociales de la conservación, colaboración, empoderamiento, participación, pérdida de autoridad, posicionamiento, Sur Global

## Abstract

Conservationists increasingly position conservation that is mutually beneficial to people and biodiversity on the promise of empowerment of people through participatory discourse, metrics, processes, and outcomes. Empowerment represents multidimensional concepts and theories that permeate the interlinking levels of power, from the psychological to the political, and social scales in which conservation operates. The multifaceted nature of empowerment makes it challenging to understand, pursue, and evaluate as a central philosophical commitment and goal‐oriented practice in conservation. Moreover, definitional and methodological uncertainty may disempower interested and affected groups because they can foster conceptual assumptions that reinforce institutionalized barriers to systemic changes. Despite these complexities, there are no targeted reviews of empowerment in conservation. We conducted a scoping review of the conservation literature to synthesize the meanings and uses of *empowerment* in the field. We reviewed 121 of the most cited conservation articles that invoked or assessed empowerment from 1992 to 2017 to document geographic, conceptual, and methodological trends in the scales and theories of empowerment deployed by conservationists. Research claiming or assessing empowerment through conservation often focused on communities in the Global South. Most studies relied on qualitative and mixed methods (78%) collected largely from male or non‐Indigenous participants. Few studies (30%) defined the 20 types of empowerment they referenced. Fewer studies (3%) applied empowerment theories in their work. Our findings show that empowerment discourse of local and Indigenous communities permeates the discourse of people‐centered conservation. Yet, overreliance on empowerment's rhetorical promise and minimal engagement with theory (e.g., postcolonial theory) risks disempowering people by obscuring empowerment's foundational value to conservation and communities and oversimplifying the complex realities of people‐centered conservation. Lasting change could come from more meaningful engagement with empowerment, including coproducing definitions and measures with and for disempowered social groups to tackle widespread power disparities in conservation today.

## Abstract (es)

El alcance del empoderamiento para la conservación y las comunidades

**Resumen:**

Con frecuencia los conservacionistas posicionan a la conservación como benéfica para las personas y la biodiversidad mediante discursos, medidas, procesos y resultados participativos que prometen el empoderamiento de la gente. El empoderamiento representa conceptos y teorías multidimensionales que permean los niveles interconectados de poder, desde el psicológico al político, y las escalas sociales en las que opera la conservación. La naturaleza multifacética del empoderamiento complica que se entienda, se dé seguimiento y se evalúe como un compromiso filosófico central y una práctica orientada hacia las metas dentro de la conservación. Además, la incertidumbre metodológica y de definición pueden restar autoridad a los grupos interesados o afectados pues pueden promover suposiciones conceptuales que refuerzan las barreras institucionales de los cambios sistémicos. A pesar de estas complejidades, no existen revisiones focalizadas del empoderamiento en la conservación. Realizamos una revisión de alcance de la literatura de conservación para sintetizar los significados y usos de la palabra empoderamiento en este campo. Revisamos 121 de los artículos sobre conservación más citados que invocaron o evaluaron el empoderamiento entre 1992 y 2017 para documentar las tendencias geográficas, conceptuales y metodológicas en las escalas y teorías del empoderamiento usadas por los conservacionistas. La mayoría de los artículos que afirmaban o evaluaban el empoderamiento por medio de la conservación se enfocaron en comunidades del Sur Global. La mayoría de los estudios dependieron de métodos cualitativos y mixtos (78%) tomados principalmente de participantes masculinos o no indígenas. Pocos estudios (30%) definieron los 20 tipos de empoderamiento que referenciaron. Todavía menos estudios (3%) aplicaron las teorías de empoderamiento a su trabajo. Nuestros descubrimientos muestran que el discurso de empoderamiento de las comunidades locales e indígenas permea el discurso de la conservación centrada en la gente. Sin embargo, depender en exceso de la promesa retórica del empoderamiento e involucrarse en lo mínimo con la teoría (p. ej.: teoría postcolonial) arriesga que la gente se pierda autoridad al oscurecer el valor fundamental que tiene el empoderamiento para la conservación y las comunidades y simplificar sobremanera las realidades complejas de la conservación centrada en las personas. El cambio duradero podría venir de involucrarse de forma más significativa con el empoderamiento, lo que incluye la coproducción de definiciones y medidas con y para los grupos sociales no empoderados para resolver la disparidad de poder que existe hoy en día en la conservación.

## INTRODUCTION

Biodiversity conservation was founded as a mission‐driven discipline to slow biodiversity loss (Soulé, 1985). Yet, this mission and its values continue to shift with evolving perceptions of the relationships among people, nature, and conservation itself (Ferraro et al., 2023; Mace, 2014; Noss, 1999; Saif et al., 2022). These relationships take many forms in changing social, political, and economic structures, which present people and nature as independent, interdependent, or in opposition to one another. For instance, centuries of colonial, preservation‐oriented conservation discourse and approaches evicted and erased Indigenous and local peoples from nature (Brockington & Igoe, 2006; Dawson et al., 2023). Other framings of conservation directly position humanity's survival on the relationships, values, and ways of knowing we ascribe to biodiversity (Maffi, 2005; Pascual et al., 2023). Within these relationships, “all conservation actions are bound up with the exercise of power” (Shackleton et al., 2023). The framing and purpose of conservation therefore determine its power in research and practice, which allows conservationists to choose how power is portrayed in conservation, the social and environmental problems they seek to address, and the communities they aim to empower (Adams et al., 2004; Carpenter, 2020; Sandbrook, 2017). This has led to decades of concerns over power imbalances between conservationists and community partners, who benefits or is harmed by conservation interventions, and how empowerment and disempowerment mediate these issues (Adams & Hutton, 2007; Brosius et al., 1998; Dressler et al., 2010).

Interest in empowerment in conservation and natural resource management has grown substantially since the 1960s (Table 1) and frequently responds to the predominant conservation framing and social movements of the time. Much of 20th century conservation was frequently criticized for separating people from nature through exclusion of local communities’ needs, values, and knowledge systems (Dressler et al., 2010; West et al., 2006). These critiques were met with calls from Indigenous Peoples and scholars of conservation, anthropology, and development to advance and critically assess the empowerment of Indigenous Peoples and local communities through sustainable development mechanisms, community‐based conservation, and participatory research and management approaches (Cooke & Kothari, 2001; Peluso, 1992; Stevens, 1997; Western & Wright, 1994). Calls to action also paralleled widespread appeals for empowerment emerging from global social movements and the social sciences (Batliwali, 2010; McLaughlin, 2016; Rowlands, 1997). Examples of paths to empowerment included poverty alleviation, alternative livelihoods, cultural recognition (e.g., local and traditional knowledge systems), collective action, resource‐use and land rights, and inclusive decision‐making in protected area (PA) planning and implementation (Berkes, 1989; Chambers, 1983; Ostrom, 1990; Pimbert & Pretty, 1995)—all of which remain cornerstones of people‐centered conservation (Murphree, 2009; Stevens, 1997). As a result, empowerment advanced from a popular, critiqued concept in conservation to a central tenet of effective people‐centered and rights‐based conservation (Brown & Rosendo, 2000; Cooke et al., 2022; Dawson et al., 2021; Murphree, 2009; Oldekop et al., 2016; Western & Wright, 1994). Empowerment definitions, dimensions, metrics, and outcomes have since proliferated in conservation, sustainable development, and the social sciences (Hennink et al., 2012; Hur, 2006; Ibrahim & Alkire, 2007; Petriello et al., 2021; Raj et al., 2022; Scheyvens, 1999).

**TABLE 1:** Percentage of Google Scholar search results linking the search terms “*empowerment*” to “*conservation*” or “*natural resource management*” from 1960 to 2023 (10 August 2023).

Period	“*Conservation*” OR “*natural resource management*” AND “*empowerment*”	“*Conservation*” OR “*natural resource management*”	Search results focused on “*empowerment*” (%)
2020–August 2023	47,500	1,030,000	4.6
2010–2019	60,800	1,800,000	3.4
2000–2009	30,100	1,840,000	1.6
1990–1999	14,500	1,480,000	1.0
1980–1989	948	814,000	0.1
1970–1979	138	438,000	0.032
1960–1969	50	198,000	0.025

Empowerment is broadly described as the process and outcome of addressing disempowerment, whereas disempowerment refers to the exclusion or absence of power, resources, and decision‐making capacity due to marginalization, disenfranchisement, and other structural, institutional, and discursive constraints (Cumming et al., 2008; Noe & Kangalawe, 2015; Singh & Titi, 1995). Because power in conservation is multidimensional and multiscalar (for comprehensive reviews, see Avelino, 2021; Carpenter, 2020; Shackleton et al., 2023), empowerment and disempowerment intersect all scales, geographies, and relationships in conservation. Petriello et al. (2021) recently identified 17 types of empowerment with varied definitions or descriptions, including community, legal, political, psychological, and women's empowerment. Other forms of empowerment, including Indigenous and women's empowerment, are also increasingly gaining prominence as conservation aims to redress systemic colonial practices, foster equitable research relations, and support local self‐determination by recognizing and integrating diverse knowledge systems into conservation planning, knowledge production, and governance (Dawson et al., 2021; Dockry, 2020; Ens et al., 2022; Ellam Yua et al., 2022). Empowerment has remained a commonplace domain of conservation and development assessments for decades (Berkes & Adhikari, 2006; Buffa et al., 2023; Chambers, 1994; McKinnon et al., 2016; Witinok‐Huber & Radil, 2021). Given its conceptual diversity, many conservationists position empowerment as an antidote to disempowerment from business‐as‐usual models of top‐down conservation or a principal *social* justification and leverage point for participatory and inclusive conservation and management (Cebrián‐Piqueras et al., 2023; Danielsen et al., 2022, 2021).

Yet, empowerment is one of many social science concepts that is often imprecisely or ambiguously used to institute and support people‐centered conservation actions, including autonomy, agency, decolonization, equity, Indigeneity, power, privilege, and trust (de Gracia, 2021; Friedman et al., 2018; Saif et al., 2022; Stirling & Burgman, 2021). This indicates a potential crisis in the “representational rhetorics” (West, 2016) of conservation that shape how conservationists and communities see and portray themselves and each other. In response, researchers are problematizing, proposing, and pursuing meaningful engagement with these concepts, including empowerment through emergent conservation approaches, such as knowledge co‐production (Zurba et al., 2022), compassionate conservation (Ferraro et al., 2023), new conservation science (Petriello & Wallen, 2015), and connected conservation (Carmenta et al., 2023).

*Empowerment* in conservation represents this potential crisis of rhetoric. It is a “fulsomely positive” buzzword (Cornwall & Brock, 2005, p. 1043) and generic term (Brosius et al., 1998) with multiple definitions, theories, and dimensions of relevance to conservation (Constantino et al., 2012). This uncertainty has generated some skepticism and caution around empowerment in conservation and development (Cooke & Kothari, 2001; Little, 1994; Petriello et al., 2021). Some scholars point to it as an important element of conservation success (Berkes, 2004; Dawson et al., 2021) and a key philosophical commitment of conservation and environmental management (Cooke et al., 2022; Murphree, 2009).

These contested perceptions and the institutional architecture around them challenge the ethical and accurate use of empowerment in conservation. For instance, conservation is deeply entangled with neocolonial capitalist systems, which may mold the limits and possibilities of empowerment (Brockington & Duffy, 2011; Büscher & Fletcher, 2020; Mbaria & Ogada, 2016). As a result, researchers may disproportionately focus empowerment efforts in communities they perceive as disempowered, such as Global South communities, based on incomplete understanding of the complexities of empowerment or an assumed need of community empowerment tied to prevailing hegemonic conservation discourse (e.g., Brown, 2002; Dressler et al., 2010). In turn, empowerment assessments may generate more risks for communities if built on restricted knowledge of the theories and contextual factors mediating different forms of empowerment, such as the interplay between agency and institutional context (Alsop et al., 2006; Narayan, 2005). These risks include claiming empowerment without support, misrepresenting the social or cognitive scales of empowerment, igniting sociopolitical tensions, reproducing existing power imbalances, and disempowering conservation researchers, governments, and communities alike (Almudi & Berkes, 2010; Dressler et al., 2010; Nayak, 2021; Twyman, 2000)—all of which may reinforce the role of conservation in maintaining and sustaining neocolonial extractive structures.

Despite its importance, there are no extensive reviews of the meanings, measurements, and conceptualization of empowerment in the biodiversity conservation literature (but see Petriello et al., 2021; Raj et al., 2022). To understand how conservationists use empowerment in conservation, including spatial, methodological, and theoretical trends, we reviewed 121 of the most cited conservation studies published between 1992 and 2017 that invoked, claimed, or used empowerment in its many forms. We consider conservationists researchers and practitioners whose actions foster good relations with nature through conservation principles and values, of any bent (Ferraro et al., 2023; Sandbrook, 2015). We also considered the history of power and empowerment in conservation and our positionality.

## BACKGROUND

### History of power, empowerment, and conservation

To understand how power has become central to conservation today, one must understand the historical processes of colonization of people and resources. Some scholars point to the doctrine of discovery as a key moment in the global expansion of the European Empire whereby the Catholic Church bifurcated the globe between Spain and Portugal to divvy up domination of people, land, and resources (e.g., Kepe, 2023; Miller, 2019). Through early and extensive *land grabs*, key processes were set in motion that enabled the development of contemporary conservation. As the global power of the Catholic Church was replaced with the power of the nation‐state in the mid‐1800s, modern processes of resource management were developed to maximize profits from natural resources (Barton, 2002). For example, European leaders drew territorial lines across Africa during the new scramble for the continent at the Berlin Conference in 1884–1885, which led to the growth of Western empires and the impoverishment of African nations (Kepe, 2023).

In 1872, a new conservation movement was born when the United States Congress established Yellowstone National Park, which launched a conservation model often described as fortress conservation due to the forced removal of Indigenous and local peoples in the name of conservation (Brockington, 2004). Concurrently, the social sciences (e.g., anthropological thought rooted in Darwinian notions of human evolution toward godliness) played important roles in the justification for the expansion of colonial ideology that separated people from nature (Ake, 1982; Marks, 2012; Rashkow, 2014).

As PAs became popular conservation tools, so, too, did rational management of natural resources. By the mid‐20th century, these power‐driven systems had established managerial control over half of the world's forests and their extractive wealth (Barton, 2002). Combined, these philosophies were wielded by the elite to justify colonial dominance over local systems of land use that were marginalized as destructive, unsophisticated, and unsustainable (Berkes, 2021).

Alongside the emergence of the modern environmental movement—a movement founded on the use of PAs and science to preserve nature—empowerment entered the zeitgeist starting in the 1960s and 1970s to center questions of social outcomes through power (Berkes, 2021; Foucault, 1980; Freire, 1970). This led scholars across disciplines to engage with participation and empowerment decades thereafter (Chambers, 1983; Conger & Kanungo, 1988; Friedman, 1992; Fung, 2003; Rappaport, 1987; Solomon, 1976). By the 1970s, conservationists also took hold of the ideas of empowerment as natural resource and sustainable development programs sought to recenter people within joint environmental and poverty alleviation schemes following decades of human–nature decoupling (Ghai, 1994). As the so‐called third world debt crisis rippled across developing countries in the 1980s, funders could no longer ignore the role of impoverished communities in conservation (Kahn & McDonald, 1995). Subsequent programs evolved into integrated conservation and development projects (ICDP), community‐based natural resource management (CBNRM), community‐based conservation (CBC), and comanagement approaches, which revealed that rural, resource‐dependent people often have communal rules governing resource use and are the first line of defense against resource overexploitation or degradation (Redmore et al., 2017). Empowerment of rural, resource‐dependent people became critical to conservation objectives as interest in participatory, community‐centered philosophies and methods reshaped conservation (Western & Wright, 1994).

Efforts to resituate local peoples as environmental stewards showed power, and by consequence empowerment, was central to community‐based management approaches (Berkes, 2004; Ellis & Biggs, 2001; Ostrom, 1990). Thereafter, failure to deliver on expectations of empowerment via community‐based poverty reduction, sustainable development, and wildlife management produced pushback about the role of people in conservation. In turn, scholars sought to identify whether and how empowerment can be a fundamental motivation and force underlying community‐based conservation and development (Kellert et al., 2000; Twyman, 2000). For example, comanagement approaches emerged as potential paths toward empowerment (Berkes, 1995) that also risked failure when they did not account for the complexities of empowerment (Brown, 2002). Others similarly showed that attempts to empower communities through participation or devolution of governance rights alone rarely redistributed power and even reinforced power imbalances (Kellert et al., 2000; Kull, 2002; Twyman, 2000).

As questions of human well‐being become inextricably linked to conservation, many social science disciplines are challenged to unravel the colonial underpinnings of their intellectual traditions in “a struggle that constantly renegotiates the balance of domination and resistance” (Pels, 1997, p. 164). In particular, work from political ecologists that centers power as a key driver of environmental outcomes has pushed for more empowerment of local, rural, and Indigenous communities toward the end of social justice (e.g., Forsyth, 2008; Robbins, 2012). Given that power is inherent to conservation, “[t]he lens through which environmental problems are constituted and projected inevitably assigns specific causations and empowers and disempowers different actors” (Peet et al., 2010, p. 37). In other words, empowerment and disempowerment are coconstituted alongside conservation.

### Definitions and domains of power and empowerment in conservation

Increased interest in understanding linkages between empowerment and conservation has been met with some challenges as evidence of its impact falls short. Hence, we aimed to understand how others define and operationalize empowerment in conservation amid a panoply of interdisciplinary empowerment definitions, domains, and theories (Hennink et al., 2012; Hur, 2006; Raj et al., 2022). We left *empowerment* undefined yet considered diverse conceptualizations of power to determine how and why scholars may invoke diverse conceptualizations of empowerment.

Conservation actors use and benefit from different forces to shift and reallocate power in different ways, encompassing neoliberal practices, decentralization, territorialization, and even decolonization. Power is widely perceived as an instrument of domination in conservation and development (Carpenter, 2020; Raik et al., 2008), though those who wield it are not always aware they hold disproportionate power (Rowlands, 1995). Social and political theory are replete with diverse definitions of power, and a recent review highlights that power in conservation can be grouped into 4 interrelated categories: actor‐centered power, institutional power, structural power, and discursive power (Shackleton et al., 2023).

Actor‐entered power is largely considered an expression of agency or “the (in)capacity of actors to mobilize resources and institutions to achieve a goal” (Avelino, 2017, p. 507). Power, in this case, is understood to be centralized in individual actors motivated by self‐interest alone, meaning that power gained by one actor is power taken away from another actor (Raik et al., 2008). In other words, actor‐centered power can be conceptualized as a finite resource. This conception of power can minimize peripheral actors or institutions that may ultimately wield more control over outcomes while it distorts the many ways in which power can be generative—and more empowering—when actors join forces (e.g., actors forming cooperatives to resist neoliberal market policies) (Fisher et al., 2020).

Institutional power centers the ways formal and informal institutions shape on‐the‐ground outcomes across multiple scales, including at the individual, community, and societal levels (Adger et al., 2005; Ostrom, 1990). Institutional power in conservation has largely grown from the body of work showing that power operates within and across scales in social–ecological systems (Adger et al., 2005). This may apply to local communities with informal and legitimate yet unsanctioned power, and governments with formal and sanctioned power that is seen by communities as illegitimate (e.g., Kashwan, 2016; Song & M'Gonigle, 2001). Institutions may ratchet up their power in a metaphorical arms race that highlights that power is not theorized as a zero‐sum game in this context (Cruikshank, 1999; Kabeer, 1999).

Theories of structural power have grown out of the Marxist traditions, whereby power is socially produced and limits how people access material and social capital through racial, class, gender, and other historically rooted contexts. Conservation often perpetuates contextual inequalities around actors’ ability to exercise power (Shackleton et al., 2023), such as through elite capture of conservation resources and benefits (Agarwala & Ginsberg, 2017). Critics of the structural perspective argue that the presumed absence of individual or group agency can mediate a sense of helplessness when structures are assumed to hold all power (e.g., Salomon et al., 2017). Conversely, structural power can also be leveraged to catalyze social change (Avelino, 2021). In fact, scholars now widely understand that people can empower each other through collective action to overcome structural power imbalances linked to environmental problems (e.g., when women and men collectively organize to improve gender parity in fisheries [Torre et al., 2019]).

Discursive power can be diffuse and difficult to locate, or present even when it appears absent, because it takes many forms and works through many contextual channels, such as everyday practice, values, knowledge, and social relations (Foucault, 1980). In conservation, discursive power often manifests when powerful conservation actors (nongovernmental organizations, governments) advance select perspectives and discourses around the norms, values, and success of participatory conservation (Bixler et al., 2015). As a result, discursive power is difficult to study or influence, leading some to question whether everyday people can have true agency in this context precisely because conservation discourse can transform people into environmental subjects without capacity to decide what counts and who matters (Carpenter, 2020). In contexts where people become environmental subjects, discursive power can disempower local actors and generate windows into multiple interrelated forms of power (e.g., institutional power [Usop et al., 2022]). For example, analyses of discursive power through a political ecology lens can reveal historical power relations that add explanatory depth to studies of power and knowledge (Robbins, 2006).

The above forms of power manifest in different ways from empowering to disempowering contexts. Avelino (2021) describes this spectrum as “power as enabling versus power as constraining” (p. 12). Similarly, Mudliar and Koontz's (2021) relational power framework counterbalances power as domination (Lukes, 2005) with power as empowerment (Allen, 1999). Power as domination—the most invoked form of power in conservation (Raik et al., 2008)—arises as power over others, which is widely considered coercive power that often disempowers through visible power (e.g., weighting decision‐making power toward one group), hidden power (e.g., one group is not given a choice to participate or the information they need to make an informed decision), and invisible power (e.g., encoding dominant ideas, values, and processes in mainstream institutions so that certain groups accept, even embrace, their disempowered status). Conversely, power as empowerment refers to power to (ability to act on one's agency); power with (collective power through group cooperation and learning); and power within (an agent's sense of self, identity, and confidence). The 2 forms of power—domination and empowerment—are mutually exclusive or interdependent depending on context. As such, the many forms and dimensions of power underlying empowerment and disempowerment can be observable, elusive, unidimensional, and multifaceted.

In this review, we considered empowerment a transdisciplinary and multidimensional concept. Previous reviews in wide‐ranging fields, such as development studies, urban planning, public health, community psychology, management studies, political science, and education, reveal a myriad of empowerment domains, frameworks, and methods for understanding empowerment's potential and pitfalls (Alsop et al., 2006; Arnstein, 1969; Hennink et al., 2012; McLaughlin, 2016; Wang & Burris, 1994). In recent decades, empowerment has been used across a variety of natural resource contexts in many different forms, such as psychological, community, and women's empowerment in forestry, marine conservation, fisheries management, and human–wildlife conflict scholarship (e.g., Danielsen et al., 2022; Fatem et al., 2023; Jentoft, 2005; Serfass et al., 2018). We recognize that *empowerment* is a conceptually rich term because it can be both discrete (i.e., measurable across time) and fuzzy (i.e., mean different things to different people) (Cornwall & Eade, 2010; Kabeer, 2000). We therefore did not seek to standardize how empowerment is used or defined in conservation; rather, we sought to foster epistemic humility in the use of empowerment and other widely invoked social science concepts in conservation.

## METHODS

### Positions of authors relative to this review

Empowerment is a culturally and politically derived concept nested within different geographies. This means we had to interrogate the social and relational context of our situated knowledge from our distinct social identities and professional disciplinary training in Western and predominantly White universities (Duffy et al., 2021; Muhammad et al., 2015). We are conservationists from North America working in North America, Central America, and Africa (A.P., C.M., L.B., L.R., M.A.P., S.B.), Africa with US credentials working in Africa and North America (A.S.), and South Asia with North American graduate educations working in India and North America (D.K., R.S.Y.).

Given our geographic and academic backgrounds, we perpetuate Western imperialist ideologies in our own conceptualizations of empowerment and conservation (Ake, 1982). However, our embedded work with excluded and vulnerable communities has fostered more critical ideological positions toward ourselves and empowerment within conservation projects (Barraclough & Zurba, 2023; Katju & Kyle, 2021; Madge, 2021; Papadopoulos, 2021; Petriello & Stronza, 2021; Redmore et al., 2020; Sène‐Harper et al., 2019; Yalamala et al., 2023; Zurba, Boyd, et al., 2022). For this review, we applied a critical emancipatory perspective, recognizing that conservation and human rights are fundamentally intertwined (Moon & Blackman, 2014). Yet, we participate in dominant models of funding and research that do not always align with local and Indigenous community priorities. Our professional activities may therefore inhibit empowerment outcomes by reinforcing institutionalized power imbalances (Wilmsen & Krishnaswamy, 2008). These tensions led us to question not only how conservation deploys empowerment, but also our own positions as active participants in a field that is as likely to disempower as it is to empower (Cooke & Kothari, 2001; Dawson et al., 2023; Noe & Kangalawe, 2015).

### Literature review

**FIGURE 1:**
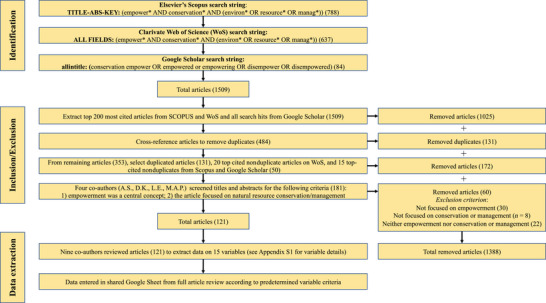
Literature review structure and criteria used to identify the types, measures, geographies, and theories of empowerment in 121 conservation studies published from 1992 to 2017.

We conducted a literature review in March 2019 to assess the meanings, measurements, and outcomes of empowerment in a sample of the biodiversity conservation and natural resource management literature. We conducted our review in 3 stages (Figure 1). First, we used 12 search strings comprising variations of 7 keywords related to empowerment and conservation with Boolean operators in 3 academic databases. General searches produced results ranging from approximately 14,000 to 142,000 articles. We narrowed the searches to titles, abstracts, and keywords in Scopus and Google Scholar. We also conducted a broader search in Web of Science (WoS) due to small variations in search results across different search fields, such as omissions of key studies (e.g., Reed, 2008).

Second, we extracted the top‐cited articles from each search engine, removed duplicates, and then drew from the 20 top‐cited nonduplicates to reduce the 1509 articles located in the identification phase. Our focus on the most cited articles meaningfully constrained our review for logistic purposes. It also best captured the literature that conservation scientists will find and are drawing from to explore empowerment in their work. However, the use of citation rates and the review date also excluded recent articles on empowerment in conservation that received fewer citations. This included articles published within 2 years of the review date (e.g., Engen & Hausner, 2018) and more recent conservation scholarship on empowerment and power (e.g., Nuno et al., 2021). This process produced 181 articles.

Third, A.S., D.K., L.R., and M.A.P. screened remaining titles and abstracts with 2 inclusion criteria (Figure 1): authors invoked claims or analyses of empowerment to support their study justifications, methods, or findings and the article focused on conservation or natural resource management problems or contexts. This produced 121 articles for the review. Articles were evenly divided among all coauthors for data extraction across 15 variables (Appendix S1). We selected the variables to determine how conservation scholars conceptually and theoretically engaged with empowerment (e.g., the theories and definitions of empowerment authors invoked); how authors assessed empowerment and from whom (e.g., measurements, study samples); how authors reported outcomes of empowerment or disempowerment; and general publication trends (e.g., geographic locations). All coauthors used the same variable descriptions, definitions, and data entry formats for data extraction (Appendix S2). Data entry formats and interpretation of data were codeveloped and cross‐referenced through iterative meetings with all authorship team members. We used descriptive statistics to reveal trends in how empowerment is defined, deployed, and discussed in the conservation literature.

## RESULTS

### Empowerment geographies

**FIGURE 2:**
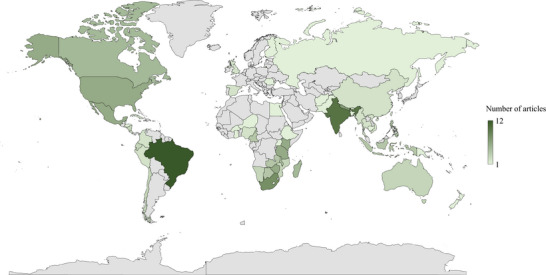
Geographic distribution of 121 empowerment studies in the conservation literature published from 1992 to 2017.

The studies in our sample included research articles analyzing primary data (41%, *n* = 50), review or overview articles (27%, *n* = 33), case studies analyzing secondary data (22%, *n* = 26), conceptual articles (8%, *n* = 10), and syntheses in journal special issues (2%, *n* = 2). One hundred (83%) of the 121 studies occurred in 62 countries (Figure 2). Twenty‐one articles (17%) were not geographically constrained or were global in scope. Regionally, most studies were conducted in southern and eastern Africa (29%, *n* = 35), Asia (26%, *n* = 31), and Latin America and the Caribbean (23%, *n* = 28). Twenty‐eight percent (*n* = 34) were conducted in North America, Europe, and Oceania combined. Brazil (10%, *n* = 12), India (8%, *n* = 10), South Africa (7%, *n* = 8), and Nepal (6%, *n* = 7) were the most represented countries. In total, 78% of the studies occurred exclusively in the Global South. Fifty‐five studies (45%) focused on 140 PAs (Appendix S3). Thirty‐eight studies (31%) analyzed or derived claims about empowerment from research in ≥2 PAs. The most studies were conducted in Annapurna Conservation Area (Nepal) (3.3%, *n* = 4) and Nanda Devi Biosphere Reserve (India) (2.5%, *n* = 3). Seven studies (13%) did not name PAs.

### Empowerment dimensions

**FIGURE 3:**
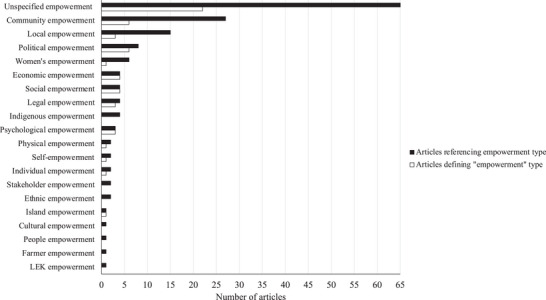
Twenty types of empowerment and the number of articles defining each type invoked in 121 empowerment studies in the conservation literature from 1992 to 2017 (unspecified empowerment, the term *empowerment* or its variants [e.g., *empowered*, *empowering*, *empower*]; community empowerment, the terms *community empowerment* or *empowerment of local communities*; local empowerment, the terms *local empowerment* or *empowerment of local people*; LEK, local ecological knowledge empowerment [Gerhardinger et al., 2009]).

Ninety‐six studies (79%) referenced, invoked, or described a total of 20 types of empowerment (Figure 3), ranging from 1 to 7 per article (mean [SD] = 1 per article [1.01]). Thirty‐six studies (30%) defined or described empowerment. Most studies (54%, *n* = 65) used the term *empowerment* or referenced empowering or empowered individuals and groups without identifying, defining, or interrogating a specific dimension of the concept. More studies referred to “community empowerment” or the “empowerment of local communities” (22%, *n* = 27) than any other discrete form of empowerment (Figure 3). Following this trend, studies defined or described unspecified empowerment and community empowerment more than other types of empowerment (Figure 3). Authors who defined empowerment referred to more types of empowerment (mean = 1.5 [1.45]) than those who did not define or describe it (mean = 0.90 [0.67]). Although 22 articles did not identify or define the type of empowerment applied, others defined it and left room for broader interpretations and changing meanings across space and time (e.g., Buch & Dixon, 2009; Dyer et al., 2014) (Table 2). Several key themes emerged from these definitions, including power, capacity‐building, control, decision‐making, resource access, self‐determination, and autonomy (Table 2).

**TABLE 2:** Definitions and descriptions of *empowerment* and corresponding empowerment domains from 36 reviewed studies (1992–2017).

Empowerment type and domain	Definition or description	Source
Unspecified empowerment, community empowerment, legal empowerment, economic empowerment, social empowerment, psychological empowerment, political empowerment	“[…] empowerment as a process, by which, people gain control over their lives, democratic participation in the life of their community and a critical understanding of their environment (Perkins & Zimmerman, 1995)” (p. 246) “[…] proponents of participatory development use the concept of empowerment as a multidimensional approach to poverty reduction arguing that if a person or group is empowered, they possess the capacity to translate their choices into desired actions and outcomes (Luttrell et al., 2009)” (p. 246).	Noe & Kangalawe, 2015
Unspecified empowerment, political empowerment, economic empowerment	“[…] a process by which people, especially poor people, are enabled to take more control over their own lives and secure a better livelihood with ownership of productive assets as one key element (Chambers, 1993:11)” (p. 202).	Brown & Rosendo, 2000
Unspecified empowerment, legal empowerment, self‐empowerment	“A properly empowered CBNRM regime is one which has legitimate boundaries, members and leadership, which has the right to plan for and use its resources, to determine the modes of that usage, benefit fully from their resources, determine the distribution of such benefits, set by‐laws for management and negotiate with other social actors” (p. 2559). Self‐empowerment: “This has taken two forms. One is evident when a local regime, through long experience, the lessons of mistakes and successes and the confidence of organisational maturity, patently becomes the senior partner in resource management. The other is confrontation, when the local regime has the weight of revenue production on its side and is willing to use its ultimate sanction—withdrawal—as a carefully orchestrated threat to gain its formal empowerment” (p. 2560).	Murphree, 2009
Unspecified empowerment, social empowerment, political empowerment, economic empowerment, psychological empowerment	“Because we deal here mainly with 'informal power that is dispersed through society and operates in all relationships' (Eyben et al. 2006:2), we adopted the broad definition of empowerment as 'a participatory, developmental process through which marginalized or oppressed individuals and groups gain greater control over their lives and environment, acquire valued resources and basic rights, and achieve important life goals and reduced societal marginalization' (Maton, 2008:5)” (p. 2).	Constantino et al., 2012
Unspecified empowerment	“Such ‘empowerment’ [for communities to solve their own water problems] includes encouraging stronger institutions based on local cultural principles leavened as necessary by concerns for equity and fair play, integrating scientific knowledge with local wisdom to identify the feasible options available to the community, and helping them to identify any external assistance they may need, for example, sources of credit or technical assistance” (p. 202).	Merrey et al., 2005
Unspecified empowerment	“a process by which people, especially poor people, are enabled to take more control over their own lives and secure a better livelihood with ownership of productive assets as one key element (Chambers, 1993, 11)” (p. 11).	Brown, 2002
Unspecified empowerment	“According to the World Bank, [empowerment] is ‘the process of increasing the assets and capabilities of individuals or groups to make purposive choices and to transform those choices into desired actions and outcomes’ (Alsop et al., 2006, p. 1). Like ‘poverty reduction’, its discursive use as a term has meaning that changes with time, culture, society, and political context” (p. 121).	Buch & Dixon, 2009
Unspecified empowerment	“Empowerment is a process in which a group develops the ability to have an input into decisions that affect their livelihoods and become more independent in defending their interests” (p. 225).	Almudi & Berkes, 2010
Unspecified empowerment	“Decision‐making power comes through empowerment, which occurs when the decentralization of resource management gives not just responsibilities, but also rights, to local communities” (p. 58).	Kull, 2002
Unspecified empowerment	“In the people‐centred perspective [on community‐based natural resource management benefits], it is the process which empowers poor people by enhancing local management capacity, increasing confidence in Indigenous potential and raising collective consciousness, as well as meeting local needs and priorities…suggest[ing] that genuine, people‐centred active or transformative participation leads to development which is truly empowering, whilst planner‐centred participation tends to be nominal with local people acting as the passive recipients of development” (p. 324).	Twyman, 2000
Unspecified empowerment	“In the realm of environmental conflicts, fundamental components may include respect, participation, and access to and control over resources (Zimmerman, 2000). In wolf recovery, empowerment can be associated with the symbolism of wolves” (p. 67).	Browne‐Nuñez et al., 2014
Unspecified empowerment	“Increased control over lives and livelihoods, including control over natural resource management, or increased land‐tenure security” (p. 135).	Oldekop et al., 2016
Unspecified empowerment	“The distribution of power and status, particularly among local peoples, including authority devolved from central and state governments to local peoples and institutions; as well as participation in decision making, sharing of control, and/or democratization” (p. 707).	Kellert et al., 2000
Unspecified empowerment	“The EDD [Empowered Deliberative Democracy] model was developed by Fung and Wright to analyze cases that 'have the potential to be radically democratic in their reliance on the participation and capacities of ordinary people, deliberative because they institute reason‐based decision making, and empowered since they attempt to tie action to discussion'' (Fung & Wright, 2001:7)” (p. 4).	Birner & Mappatoba, 2002
Unspecified empowerment	“[T]he empowerment of the [fishing] industry includes the recognition that the functions of government at the disposal of industry provide value‐added” (p. 392).	Lane & Stephenson, 2000
Unspecified empowerment	“[T]he processes in which the stakeholders learn to be active users of power and to respect the active role of other stakeholders’ empowerment” (p. 124) “In this study, we start from the notion that empowerment is a process in which a person's inner motivation increases (Conger & Kanungo, 1988; Thomas & Velthouse, 1990) and strengthens one's perceptions of self‐efficacy and a belief in performing well (Bandura, 1977). In addition, because the empowering process has its social and societal context, it helps persons to interpret their possibly problematic social positions and change them into new actor identities (Carr, 2003; Conger & Kanungo 1988; Freire, 1970)” (p. 126).	Paloniemi & Vainio, 2011
Unspecified empowerment	“[The local development and approval of natural resource rules and regulations] is an example of empowerment, defined by Chambers (1983) as ‘the process through which people, and especially poorer people, are enabled to take more control over their own lives, and secure a better livelihood, with ownership of productive assets as one key element’” (p. 1290).	Clements et al., 2010
Unspecified empowerment	“Empowerment enables people to have control and use of their own resources, set their own agendas, and work towards achieving their aspirations. In order to achieve these, they should have access to information and services, and a developed capacity to determine their own future” (p. 312).	Devendra & Leng, 2011
Unspecified empowerment	Empowerment is inferred to mean “increased […] resource management capacity, field experience and naturalist skills, and […] motivation and pride in […] work” (p. 2529).	Danielsen et al., 2005
Community empowerment, social empowerment, political empowerment, psychological empowerment, gender empowerment	“To empower those who are disadvantaged means developing confidence and changing attitudes and behaviors that allow individuals and groups to alter the power differentials in their community, in effect creating new spaces of control (Nelson & Wright, 1995). This involves working to improve empowerment at three levels: the psychological (enhancing self‐esteem among community members); the social (improving community cohesion); and the political (improving political structure that fairly represents the needs and interests of all community groups) (Friedman, 1992)” (p. 231).	Torell et al., 2010
Community empowerment	First sentence of “Community empowerment” section: “Another impact of participatory processes on sustainability indicator selection has been to increase community capacity to manage the environment in all three regions” (p. 124).	Fraser et al., 2006
Community empowerment	“[…] it [an assets‐based approach in conservation] empowers local people to participate in conservation efforts. Governments are then more likely to respond favorably to community demands. This can then strengthen local peoples’ capacity to create sustainable livelihoods and improve well‐being” (p. 1).	Wali et al., 2017
Community empowerment	“Empowerment infers the rebalancing of power to disenfranchised stakeholder groups through awareness raising or education (Potter et al., 1999), but can, and should, be viewed from multiple perspectives (Twyman et al., 2001)” (p. 139). “Empowerment was clear when project participants had been given the authority to make decisions and were able to justify these” (p. 144).	Dyer et al., 2014
Community empowerment	“The central element of co‐management is the empowerment of the community of local resource users (e.g., fishers) by enabling them to participate, control and influence institutional decisions affecting their lives [Jentoft, 2005; Pomeroy and Viswanathan, 2003; Wilson et al., 2006]” (p. 818).	Maliao et al., 2009
Local empowerment	“About 30% of projects [Fauna & Flora International Biodiversity & Human Needs Programme projects from 2004−2007] focused on influencing structures and processes (building organizational capacity and developing appropriate policies), and a similar proportion [9 of 34] aimed to increase local empowerment, mainly through participatory approaches to increase local engagement in conservation decision making” (p. 542).	Walpole & Wilder, 2008
Local empowerment, individual empowerment	“[…] foster local empowerment at the individual and collective levels, through training, career development and decision‐making sharing (social sustainability)” (p. 223).	Lapeyre, 2011
Local empowerment	“It [the co‐ordinated research management approach] empowers the local people directly affected by the establishment of the protected ecosystem to solve their own problems in a voluntary, non‐regulatory manner thereby strengthening the local economy and social well‐being as well as the natural resources” (p. 98).	Gbadegesin & Ayileka, 2000
Empowerment of local people	“Many of the [Integrated Natural Resource Management] guidelines involve empowering local stakeholders directly, by allowing them to be heard and through amplifying their voices, and indirectly, by building their capacity to be more effective implementers of their own development and conservation efforts.”	Frost et al., 2006
Political empowerment	“Political empowerment refers to people's ability to participate in and influence decision making processes that affect their lives (UNDP et al., 2005)” (p. 1238).	Stephanson & Mascia, 2014
Political empowerment	“During the Maijuna mapping project, it became increasingly clear that the act and process of map making was politically empowering to the Maijuna, as highlighted by the fact that they would frequently engage in conversations about the political importance of the maps that they were producing” (p. 22).	Gilmore & Young, 2012
Economic empowerment	“Introduction of people‐owned and people‐managed credit and savings enhanced their sense of ownership, self‐identity and confidence and provided economic empowerment in the form of capacities required for engaging in new enterprises” (p. 91).	Rahman & Begum, 2010
Legal empowerment	“ The UN‐sponsored Commission on the Legal Empowerment of the Poor defines legal empowerment as 'the process by which the poor are protected and enabled to use the law to advance their rights in the face of claims by both the public and the private sector' (CLEP, 2008, p. 38)” (p. 19).	Boudreaux & Nelson, 2011
Physical and social empowerment, island empowerment	“Molocaboc fishers should be included in the monitoring activities, through both physical and social empowerment (for example, monitoring vessels and a strong decision‐making voice), combined with outreach or extension activities by the SMR[Sagay Marine Reserve] management staff to clearly define the needs and solutions envisioned by the Molocaboc Island residents” (p. 145).	Webb et al., 2004
Unspecified, unknown	“For instance, *empowered* stakeholders can decide about the ecosystem services supplied and regulate access to them, negatively affecting non‐empowered stakeholders by reducing their ability to access ecosystem services” (emphasis added, p. 2).	Felipe‐Lucia et al., 2015
Unspecified, unknown	“Involvement of rangers in the research [Marchinbar Island case study in Australia] has led to them understanding western scientific perspectives on the species’ significance. This has *empowered* them to advocate within their community for the removal of feral dogs” (emphasis added, p. 573).	Garnett et al., 2009
Unspecified, unknown	“Kok et al. (2007) and Walz et al. (2007) argue that stakeholder engagement in scenario development *may empower* those involved, through the co‐generation of knowledge with researchers and increasing participants’ capacity to use this knowledge” (emphasis added, p. 347). “The framework we have presented and applied emphasises the need to combine local and scientific knowledge. It is argued that the integration of information from stakeholders with evidence from research has the potential to *empower* stakeholders, and develop more consistent, detailed and precise (though of course, not necessarily accurate) scenarios than could be developed from local or scientific knowledge alone” (emphasis added, p. 359).	Reed et al., 2013

*Note*: Unspecified empowerment denotes the general use of the term *empowerment* or its variants (e.g., *empowered*, *empowering*) by the author.

### Empowerment assessments

Ninety‐five studies (79%) analyzed empowerment with qualitative (47%) or mixed methods (31%). Eleven (9%) studies predominantly relied on quantitative methods. In addition, 13 (11%) did not collect primary data or directly evaluate empowerment. Case studies, semistructured interviews, focus groups, and participant observation were the most commonly deployed methods, whereas visual participatory methods, such as photovoice and participatory mapping, were less common (Appendix S4). Mixed methods and quantitative studies numerically assessed empowerment with social and ecological indicators in the form of Likert‐type questions (e.g., empowerment scales [Constantino et al., 2012]), frequency counts of categorical or ranked responses to empowerment‐themed measures, biophysical proxies for empowerment (e.g., local capacity to measure biomass [Danielsen et al., 2011]), and self‐assessments of empowerment (e.g., “How are employment relationships in this [public tourism] partnership, when compared with relations in a private project?” [Lapeyre, 2011, p. 227]) (8%, *n* = 10). These data were largely analyzed at the levels of case studies, households, and participants (Appendix S5). Analyses from only 29 articles (24%) drew data from or made claims about the empowerment or disempowerment of 47 Indigenous groups, such as the Haida Nation, Kaxinawá, and Zoque peoples (Appendix S6). Fewer articles (*n* = 11, 9%) reported participants’ genders, which were skewed toward men (mean [SD] = 158 [218]) over women (mean = 96 [149.25]).

Of the 121 reviewed studies, 15 (12%) engaged with at least one theory, model, or framework to directly interpret empowerment or indirectly derive inferences about the concept. Twelve of these 15 studies engaged with empowerment through 12 distinct theories, models, or frameworks for natural resource management and stakeholder engagement (Table 3). Three studies (3%) drew on 3 empowerment‐centered theories, models, or frameworks: Fung and Wright's (2001) empowered deliberative democracy model, Longwe's (2002) framework for women's empowerment, and Scheyvens’ (2000) model on women's empowerment in ecotourism (Table 3).

**TABLE 3:** Names, descriptions, or justifications and sources of 15 theories and frameworks from 15 out of 121 papers (12%) used to directly or indirectly investigate empowerment.

Theory, model, or framework	Description or justification	Source
Fung and Wright's (2001) Empowered Deliberative Democracy (EDD) model	“The EDD model was developed by Fung and Wright to analyze cases that “have the potential to be radically democratic in their reliance on the participation and capacities of ordinary people, deliberative because they institute reason‐based decision making, and empowered since they attempt to tie action to discussion” (Fung & Wright, 2001: 7)” (p. 4).	Birner & Mappatoba, 2002; Thomas, 2001
McDermott et al.'s (2013) equity framework	“The framework has three core dimensions: distributive, procedural and contextual equity, which together describe the substantive content (the what) of equity. Three additional parameters are necessary for a complete analysis of equity in a given intervention: its goals in relation to equity (why), the target groups, or social scale, of equity (who) and ‘how’ the goals, targets and content of equity were decided upon in the first place (cf. Fraser, 2009)” (McDermott et al., 2013, p. 17).	McDermott, 2013
Longwe's (2002) framework for women's empowerment	“The first model, Longwe's Empowerment Framework, is designed to understand how women's participation in ecotourism projects, as development projects, may or may not promote levels of empowerment for women, moving from welfare, access and conscientization to participation and control. This analytical model can be used to situate women's participation in an ecotourism project in relation to each of the levels, and provides a direction for movement up the framework towards greater equality and empowerment for women” (p. 118).	Tran & Walter, 2014
Scheyvens' (2000) model on women's empowerment in ecotourism	“Scheyvens's (2000) framework of empowerment for women, puts not women, but women‐in‐community, at the center of its analysis […] the unit of analysis in Scheyvens's model is first, the wider community involved in ecotourism, and second, women within this community […] [it] captures much of the complexity not only of gender, but also of class, ethnicity, and social and community structures. On the other hand, women are only one of many possible marginalized groups in the model and may be de‐emphasized in the wider concerns over other types of oppression” (p. 119).	Tran & Walter, 2014
Framework of rationales for engaging with poverty	“Numerous theoretical frameworks to categorize poverty‐conservation relationships exist (Adams et al., 2004; Nadkarni, 2000; Roe & Elliot, 2005). These outline a range of rationales for conservationists to engage with the poor, which in turn dictate the approaches taken by practitioners and the likely outcomes, not all of which will necessarily reduce poverty (Table 1). This is not always understood, particularly amongst conservation biologists. To achieve greater clarity we must disentangle the links by exploring how the rationales in these conceptual frameworks are played out in practice, and which elements of poverty are at the fore (Robinson, 2006; Sunderlin et al., 2005)” (Walpole & Wilder, 2008, p. 540; table 1, p. 541).	Walpole & Wilder, 2008
Community‐based natural resource management (CBNRM) and participatory frameworks	“Community‐based natural resource management frameworks have at times been seen as solely conservation projects, and as such have rarely been critically evaluated in terms of development theory, which would acknowledge the power and positionality of the different stakeholders. There is a need to examine these programmes in terms of local understandings and opinions of community‐based natural resource management initiatives, and local relationships with the environment. The extent to which these programmes have been shaped by local priorities or government agendas will reflect the power relationships involved and the balance between conservation and development objectives within the programmes. Central to the ethos of community‐based natural resource management is the ‘participation’ of local people and their ‘empowerment’ through the development process” (p. 324).	Twyman, 2000
Fisheries comanagement research framework	“All studies included in the meta‐analyses used the fisheries co‐ management research framework developed by the Worldwide Collaborative Research Project on Fisheries Co‐management (WCRPFC) […] The fisheries co‐management research framework employed a common set of indicators based on the perceptions of the local fishers to evaluate the performance of fisheries co‐management programs […]” (pp. 818–819).	Maliao et al., 2009
Framework of actions in support of integrated natural resource management (INRM)	“Integrated natural resource management (INRM) is an approach to managing resources sustainably by helping resource users, managers, and other stakeholders accomplish their different goals by consciously taking into account, and aiming to reconcile and synergize, their various interests, attitudes, and actions […] As such, INRM is interdisciplinary and multiscaled, encompassing different but linked levels of social and biophysical organization. It is responsive to different histories, sites, and circumstances, and is intended to integrate varied and complex sets of knowledge into a common framework for analysis and action.”	Frost et al., 2006
Working model for engagement between academics and Indigenous communities	“Here, as members of a collaborative research team consisting of academic ecologists and community experts, we present a working model for engagement between academics and indigenous communities. The model is based on our past and present experiences. We start by identifying the contemporary political context in which communities and ecologists are situated in Canada and beyond. We then identify current limitations toward community engagement. Finally, we provide a framework of key principles and roles within the research process that can yield new knowledge and a mutually beneficial process by which research can occur. Although we focus on the ecological experience, we recognize that the principles, process, and limitations of community engagement could be applicable across disciplines, especially within the natural sciences.”	Adams et al., 2014
Political economy of cross‐scale linkages framework	“We argue that part of the persistence and stability of the governance system depends on the distribution of benefits from cross‐scale linkages, demonstrated by the ability of the system to command legitimacy and trust among the resource user and the governmental stakeholders. If the structure of cross‐scale linkages reduces trust then the robustness of the system is in question.”	Adger et al., 2005
Participatory forest management conceptual framework	“The framework presented in Figure 2 is used to conceptualize the factors influencing members' participation in the forest management program in southern Burkina Faso. The framework implies that participation outcomes are shaped by the structure of incentives for the members, which is affected by the context. The context, in turn, is defined by: (1) the social network system (norms, values and social capital), (2) members' socio‐economic and demographic attributes (gender, age, level of education and income, etc.) and (3) the internal and external institutional context (technical assistance and the patterns of authority that govern the forest management system)” (p. 294).	Coulibaly‐Lingani et al., 2011
Collaborative, landscape‐scale natural resource management framework	“Whereas participation encompasses a broad spectrum of engagement, notions of collaboration emphasize high levels of interaction and shared responsibility and influence on the participatory agenda and goals […] These overlaps provide flexibility within a participatory framework as levels of involvement and interaction can be adapted to suit the particular issues or actions under consideration. However, they may also give rise to uncertainty and differing expectations that could undermine the key principles of participatory governance […] Balancing these potential benefits and significant challenges requires careful consideration of where and how shared responsibility might best be implemented and sustained” (pp. 161–162).	Davies & White, 2012
Model of public opposition to neighboring parks	“Although conflicts can be characterized in many ways, this article is primarily concerned with the decisions of residents living within the immediate vicinities of national parks to actively oppose park presence, policies, or management. It draws upon original research to advance a model of individual action that describes why conflicts persist in and around protected areas and identifies potential avenues for conflict resolution” (p. 860).	Stern, 2008
Citizen science conservation behavior feedback model	“[…] we conceive of the relationship between participation in citizen science and resultant effects on personal conservation attitudes and behaviors as a cycle or feedback loop (Figure 1) in which participation itself leads to the perception of having done something positive for the environment (“greener sense of self”), thus leading to more positive attitudes towards conservation behaviors in general, reinforcing motivations that led the participants to join the project in the first place” (p. 52).	Toomey & Domroese, 2013
Community natural resource management (CNRM) model	“The main argument is that CNRM policies that ignore internal power relations attract unintended consequences that undermine achievement of conservation and social goals. Findings show that CNRM did not improve forest management, and in many cases made it worse. Both the CNRM concept and its implementation created new local elites (forest committees) who largely operated as corrupt, unaccountable ‘village bureaucracies,’ alienating communities from CNRM. Forest overharvesting and degradation, and weakening or dysfunctional institutions ensued. Rare success was associated more with good leadership qualities of village heads, especially their ability to balance the exercise of power among various social actors, than with mere adherence to procedure, rules, or design principles” (pp. 268–269).	Zulu, 2008

*Note*: McDermott (2013) was the analyzed study in this review. However, the framework description is quoted from McDermott et al. (2013) due to the length of McDermott's (2013) description.

## DISCUSSION

Echoing decades of reviews and perspectives (Berkes, 2004; Cebrián‐Piqueras et al., 2023; Kellert et al., 2000; McKinnon et al., 2016; Murphree, 2009; Oldekop et al., 2016; Western & Wright, 1994), our review illustrates that the field of conservation is committed to enacting empowerment and counteracting disempowerment. This commitment has led decades of conservation scholars and practitioners to explore 20 types of empowerment across 62 countries through increasingly diverse methods and research contexts. However, the reviewed articles suggest that the growing interest in empowerment and disempowerment remains fairly limited in geographic scope, types of empowerment, and theoretical approaches. There are several explanations for these trends and how they potentially correspond to different forms of power.

### Empowerment geographies

Empowerment research in conservation has largely centered on the Global South and PAs. Most biological and cultural diversity occurs in Global South countries, which are also experiencing some of the highest losses of biocultural diversity worldwide (Loh & Harmon, 2005; Maffi, 2005). In our sample, more studies took place in Brazil, India, and South Africa than other countries (Almudi & Berkes, 2010; Badola & Hussain, 2003; Dyer et al., 2014). These countries house biocultural hotspots and exemplary PA networks that attract significant conservation research and funding (Loh & Harmon, 2005; Waldron et al., 2013; Wilson et al., 2016). Extensive funding and attention have thus made PAs and community‐based approaches de facto conservation strategies worldwide and, therefore, central drivers of empowerment and disempowerment in conservation (Oldekop et al., 2016; McKinnon et al., 2016; Milupi et al., 2017). These countries also have high income inequality, are home to diverse Indigenous communities, and have long been targets of urbanization, industrialization, and neocolonial conservation projects that historically occurred at the expense of rural and Indigenous resource‐dependent communities (Adams, 2004; Leisering, 2021; Loh & Harmon, 2005). Similar to *equity* in conservation (Friedman et al., 2018), the geographies of *empowerment* in conservation suggest there is a strong overlap between empowerment‐related concerns, threats to biodiversity, and governance systems that are perceived as less robust.

This overlap further suggests that the needs and effects of empowerment on Global South communities, particularly those close to PAs, may be more salient to conservation researchers for 2 reasons. First, the perceived lack of capacity among Global South communities may provide justification for conservationists to levy empowerment claims or deploy empowerment projects. The perception that local and Indigenous communities lack capacity and knowledge is part of a larger global system of colonialism that privileges knowledge produced in the Global North over situated knowledge in the Global South (de Gracia, 2021; Smith, 1999). Within the colonial legacy of conservation, perceived capacity deficits have been used to justify exploitative conservation actions for decades (Adams, 2004). Implicit to this justification is that communities require capacity building due to insufficient knowledge, motivation, resources, and technical expertise (i.e., limited actor‐centered or institutional power) to change their lives, independent of the conditions that often promote disempowerment in the first place (e.g., land grabs, centralized resource control). Several descriptions of empowerment found in this review directly referenced capacity building or capacity deficits (e.g., Frost et al., 2006; Table 2). Conversely, Danielsen et al. (2022) suggested that an overemphasis on capacity sharing may compromise the integrity of data from participatory conservation. Others showed that empowerment and capacity building may be secondary goals in conservation interventions (Campbell & Vainio‐Mattila, 2003; McKinnon et al., 2016). These competing perspectives highlight that empowerment and related terms (i.e., capacity‐building) may still be difficult to disentangle from their colonial uses in conservation.

Second, the growing awareness of empowerment may be a response to colonialism in conservation, including value shifts toward counteracting ongoing Western neocolonial practices (e.g., structural power) in the Global South (Dawson et al., 2023; Kashwan et al., 2021). For example, many studies in southern Africa focused on empowerment through CBNRM (e.g., Constantino et al., 2012; Dyer et al., 2014; Napier et al., 2005). This paradigm was developed to counteract Western models of neocolonial conservation through the inclusion of local voices in decision‐making (Dressler et al., 2010). In turn, this example suggests that the dominance of studies on PAs and empowerment in this region may be directed toward redressing the colonial roots of conservation (Adams & Hutton, 2007; Brockington & Schmidt‐Soltau, 2004; Dawson et al., 2023).

Yet, these trends extend to the Global North. Zoque communities in Mexico empowered themselves through shared ethnicity, agency, and collective action (i.e., actor‐centered power) to redefine top‐down, externally funded terms and goals for participatory conservation (i.e., discursive power) (Walker et al., 2007). Authors of another study reviewed 3 community‐based conservation principles that empowered the Wemindji Cree Nation to assert their institutional power in response to external neoliberal agendas shaping conservation (Mulrennan et al., 2012). Additional examples of bottom‐up collaborative research and comanagement efforts in Canada, Europe, and the United States showcase the empowerment of Indigenous Peoples, local communities, and even managers through citizen science initiatives, community‐led research agendas, and locally defined conservation indicators (Adams et al., 2014; Fraser et al., 2006; Mulrennan et al., 2012; Toomey & Domroese, 2013). This broad geographic distribution of studies demonstrates that external, colonial pressures (e.g., corporations, colonial governments) motivate multiple types of empowerment at regional and global scales.

### Empowerment dimensions

Empowerment, disempowerment, and power permeate most scales, processes, and outcomes of conservation. Similar to the 17 types of empowerment found in previous work (Petriello et al., 2021), the 20 types of empowerment found in this review support this claim. Within this diversity of interpretations, the dominant focus on unspecified empowerment, community empowerment, and local empowerment illustrates that conservation tends to frame empowerment as a generic concept of broad relevance to all elements and scales of conservation and as a targeted concept of specific sociospatial relevance to community‐based conservation approaches.

Our results indicate that *empowerment* remains a buzzword (popular term) and fuzzword (ambiguous term) in conservation. While buzzwords and fuzzwords are common in conservation and development (Cornwall & Eade, 2010; Goldstein, 1999; Rohwer & Marris, 2021), lack of clarity around empowerment may negatively cast a “layer of discursive blur” (Büscher & Dressler, 2007, p. 596) over an increasingly important concept to conservation's mission, discourse, and success. In other words, *empowerment* and its variants are at risk of becoming uncritical and unchallenged rhetorical devices for upholding conservation narratives and realities (i.e., discursive power), also described as “representational rhetorics” (West, 2016). In describing representational rhetorics, West (2016) asserts “economic and social power bring about the ‘truth’ that is endlessly rhetorically produced and reinforced […]” (p. 7). This truth in conservation may partly infer that some conservationists reflexively invoke empowerment and related concepts (e.g., community and capacity building) for 2 reasons: to sustain proactive narratives and images of conservation and conservationists and to condense the complex realities of working with local peoples to address environmental problems. Indeed, ambiguity around the term *empowerment* may allow those in power to shape it as something one group does to another, shift attention toward (or away from) other forms of power, or even perpetuate elite capture in conservation and development initiatives (Agarwala & Ginsberg, 2017; Alsop et al., 2006; Noe & Kangalawe, 2015; Rowlands, 1997). Defaulting to general or unspecified forms of empowerment suggests that empowerment may be or become a rhetorical tool to justify or maintain the status quo of conservation with vulnerable communities. This highlights a need to maintain awareness of empowerment's “historical discursive legacy” (Avelino, 2021, p. 13) in conservation research and practice.

The substantial attention to community empowerment reflects a history of focused attention and blurry rhetoric around the term *community* in conservation (Agrawal & Gibson, 1999; Western & Wright, 1994). In our review, community empowerment appeared largely centered on institutional arrangements and participatory methods with place‐based social groups and organizations, most often Indigenous Peoples and local communities. For example, comanaged mangrove concessions in Ecuador were sources of community empowerment that granted autonomy, control, and ancestral rights (i.e., institutional power) to coastal communities and local associations (Beitl, 2011). Fraser et al. (2006) highlight how the inclusion of local communities in identifying sustainability indicators increased communities’ management capacities (e.g., knowledge mobilization, comanagement agreements) as a form of community empowerment. However, authors’ tendency to use *community empowerment* did not always engage with the rhetorical and conceptual complexity of *community* as the locus of participatory conservation and the solution to many of its social problems. This is supported by the few authors who described or defined *community empowerment*. Disproportionate attention to community empowerment may also deemphasize the significant reach of diverse forms of conservation, collective action, and empowerment that bridge community, state, and global scales, such as “organic empowerment” (see Romano [2017] for a detailed overview).

### Empowerment assessments

Empowerment metrics and theories are as multiscalar, multidimensional, and transdisciplinary as the construct of empowerment. The application of diverse methods for exploring empowerment perhaps indicates the fundamental role of transdisciplinary approaches in emphasizing power relations in complex social issues tied to conservation (Brandt et al., 2013; Knapp et al., 2019). Methodological diversity may additionally support a “logic of empowerment” (Hölsgens et al., 2023), wherein all research collaborators are motivated to pursue a plurality of data sources and worldviews to holistically design questions of empowerment across scales and disciplines. Of the few studies in our sample that deployed quantitative measures to deconstruct the complexities of empowerment, Likert‐type scales and multiple‐choice social and ecological indicators were the most common tools (Constantino et al., 2012; Paloniemi & Vainio, 2011). However, these measures were regularly paired with qualitative interpretations of empowerment contexts and drivers. Scales and one‐dimensional indicators offer informative yet simplified depictions of complex phenomena that may be easily shared with nonspecialists and community partners. They are also unlikely to capture the full extent of empowerment impacts because they may not be appropriate as stand‐alone measures of empowerment or forms of power that are incommensurate with empirical observations (i.e., discursive and structural power) (Alsop et al., 2006; Kabeer, 1999; Narayan, 2005; Shackleton et al., 2023). This rationale may explain why most reviewed studies relied on qualitative or mixed‐methods approaches to explore empowerment, such as semistructured interviews, focus groups, and qualitative case study analyses (Almudi & Berkes, 2010; Garnett et al., 2009).

Methodological pluralism did not always lead to diverse sampling or theoretical foundations. Few studies appeared to interrogate intracommunity demographics and power dynamics, such as reporting study participants’ genders or actively collaborating with Indigenous communities. This may create various forms of sampling bias or decontextualized data interpretation that reinforce increasingly hidden forms of disempowerment. Samples from the reviewed studies suggest that men may be sampled more frequently due to gendered literacy rates, roles in natural resource extraction, visibility in the community, or statuses as heads of households (Coulibaly‐Lingani et al., 2011; Groom & Harris, 2008; Napier et al., 2005). These biases may occur even when women have deeper knowledge about and more control over management of socioecological systems (James et al., 2021). Moreover, <25% of studies collaborated with Indigenous communities, representing significant gaps in knowledge about how Indigenous Peoples are navigating and resisting systems of disempowerment at the expense of ecosystems that have stewarded for millennia (Colchester, 2000). In both instances, research that fails to account for gender, ethnicity, or indigeneity may advance poorly developed understandings of conservation problems or potential solutions given that women, ethnic minority groups, and Indigenous Peoples are critical change agents and environmental stewards for conservation and development (Dawson et al., 2021; Fletcher et al., 2021; Kabeer, 1999).

Furthermore, few studies (Table 3) engaged with theories of empowerment and wider bodies of social science literature that identify gender, age, indigeneity, and ethnicity as fundamental axes of power and empowerment (Allen, 1999; Alsop et al., 2006; Longwe, 2002; Scheyvens, 2000; Smith, 1999). Of note is Tran and Walter's (2014) interpretive case study of the gendered dimensions of participation in community‐based ecotourism (CBET) in Vietnam. The authors integrated 2 of the 3 empowerment theories and frameworks from the reviewed studies to determine how 4 dimensions of women's empowerment (i.e., economic, political, psychological, social) interacted with 5 levels of empowerment (i.e., access, conscientization, control, participation, welfare). Their theory‐driven case study showed otherwise unavailable connections between dimensions and levels of power and empowerment, such as how CBET income elevates women's economic empowerment through improved financial welfare and access to investment resources (e.g., agency‐centered power). This bolstered awareness (i.e., conscientization) of gendered labor norms and rights (i.e., discursive power), which psychologically empowered women to seek leadership roles in CBET and beyond. In turn, challenging gender roles (i.e., structural power) increased women's participation, which elevated their social empowerment and political empowerment through greater control over CBET decision‐making practices and community policy reforms (i.e., institutional power). These tools and methods are valuable frameworks for decoding the multiple forces driving relationships and changes among diverse forms of power and empowerment.

Despite minimal theoretical engagement, no articles drew from postcolonial understandings of power, which may present barriers to enacting equitable conservation in the Global South. Postcolonial scholarship interrogates power within systems of domination and colonial order (Mowatt, 2021). Postcolonial theorists therefore undertake analyses that pay attention to the point and nature of disempowerment or oppression in colonial, racialized, and capitalist relations (Bright et al., 2022). For this reason, they are principally concerned with community sovereignty and self‐determination outside of imperialist systems of land and resource management (Ajl, 2021). Similarly, political ecologists interrogate how configurations of power in neocolonial systems (e.g., structural and discursive power) shape resource use and conflicts relevant for conservationists. Yet, the work of political ecologists, particularly those drawing from postcolonial theory, remains vastly overlooked in conservation projects (Carpenter, 2020). Empowerment in conservation through a postcolonial lens would foreground oppression, land tenure, sovereignty, and self‐determination in all questions related to resource management. This is critically important considering recent robust analyses showing that lands where Indigenous communities have sovereignty over resource governance play indispensable roles in global biodiversity conservation (Dawson et al., 2021; Fa et al., 2020; Garnett et al., 2018). In these ways, postcolonial theory could highlight how empowerment‐centered conservation projects can support political, decolonial, and anticolonial struggles of Indigenous and local communities (Dawson et al., 2023, 2021).

### Empowerment in conservation

Conservation is an exercise and expression of power. Power in conservation is the basis for decisions to act or not; the development and tracking of goals and metrics; the effects of human populations on the environment; and the identification of target species or systems of conservation concern. In this vein, the growing influence of empowerment in conservation discourse, metrics, and goal setting signifies that the concept is here to stay. Our review underscores that empowerment plays a dual role in conservation: it is a central tenet and a litmus test for the effectiveness of people‐centered conservation. This dual role appears to underscore the value of empowerment's diverse meanings, measures, and goals to conservation, as well as its challenges as a conceptually and theoretically varied domain of conservation assessments and prioritization. More work that explicitly examines assumptions of empowerment in conservation may be critical for fortifying the conceptual and rhetorical value of empowerment in conservation research and practice.

The overlapping problems conservationists seek to address by empowering systemically disempowered social groups are too urgent, rapidly evolving, and widespread for shallow engagement with the concept. Conservationists should resist using the rhetoric of empowerment without engaging with its multifaceted definitions, domains, and theories. This suggestion aligns with recommendations for conservationists to more meaningfully engage with equally important and imprecisely invoked concepts, such as *equity* (Friedman et al., 2018). Conservation initiatives pursuing empowerment could co‐produce meaningful definitions and measures of *empowerment*, *power*, and related constructs with local conservation partners—all of which could bridge the gap between decolonial scholarship and conservation practice (e.g., Buffa et al., 2023). This could not only catalyze key conversations about an array of people's needs and wishes, but also fundamentally transform the conservation space through the practice of equitable power sharing and empowerment‐driven co‐production (Chambers et al., 2021; Petriello et al., 2022). Combined, these steps highlight an important barrier to harnessing the potential of empowerment and minimizing the risks of disempowerment in conservation that could be addressed through the cataloging of its diverse definitions, interpretations, and measurements.

Conservationists could also pursue epistemic humility by transparently reflecting on their positionality in relation to the context, goals, and assumptions about empowerment that inform their work (e.g., Duffy et al., 2021; Pienkowski et al., 2023). Such humility could help in the reevaluation of the boundaries of power placed around knowledge in ways that recognize and embrace diverse ways of knowing and being (Moon & Pérez‐Hämmerle, 2022). Yet, empowerment is not a panacea; its relevance and forms will manifest in different ways across diverse contexts. Top‐down and externally imposed forms of empowerment, regardless of intentions, run the risk of disempowering those who have the most to gain and lose from empowerment in conservation. This risk may be magnified when social scientists or disempowered groups are peripheral to empowerment efforts in conservation contexts; when conservation failures or successes lie within researchers’ comprehension of key social science terms, such as *empowerment* and *equity*; and when both factors impede the ability to meaningfully move social theory into practice (Martin, 2020). Yet, similar to recommendations to benefit transdisciplinary approaches through engagement with critical theory (Knapp et al., 2019), these risks may be minimized with deeper engagement with empowerment frameworks and postcolonial theory. Such engagement could position future research to further examine the role of conservation discourse, research, and practice in enabling or impeding diverse forms of empowerment, including paths toward more empowering research and engagement practices. We extend Carol Carpenter's (2020) sentiments about power in conservation to empowerment in that “it is more productive to assume that conservationists are well meaning, though some of them need to learn more about [em]power[ment]” (p. 207). This may be especially critical as scholars increasingly emphasize the importance of how conservation work gets done by embracing the potential of empowerment as a core foundation of 21st‐century conservation.

## Supporting information

Supporting Information
